# Supplementary material for: Binding the Acoustic Features of an Auditory Source through Temporal Coherence
Source: Cereb Cortex Commun. 2021 Oct 6;2(4):tgab060. doi: 10.1093/texcom/tgab060 (PMC8567849; doi:10.1093/texcom/tgab060)
Supplement: Supplementary_Figures_tgab060 [file supplementary_figures_tgab060.pdf]

# **Supplementary Figures for:** Binding the Acoustic Features of an Auditory Source through Temporal Coherence

Mohsen Rezaeizadeh[1 †], Shihab Shamma[1,2 ‡]

**1** Institute for Systems Research & Department of Electrical and Computer Engineering, University of Maryland, College Park, United States

**2** Département d'études cognitive, Ecole Normale Supérieure, Paris, France

**Abstract**

Numerous studies have suggested that the perception of a target sound source can only be segregated from a complex acoustic background if the acoustic features underlying its perceptual attributes (e.g., pitch, location, and timbre) induce temporally modulated responses that are mutually correlated, and that are uncorrelated from those of other sources in the mixture. This "temporal coherence" hypothesis asserts that listening attentively to one or a subset of attributes of a target source enhances their neural responses and concomitantly enhances all other coherent responses, thus binding them together while simultaneously suppressing the incoherent responses to the background features. Here we report on EEG measurements in human subjects engaged in various sound segregation tasks that demonstrate rapid binding among the temporally coherent features of the attended source regardless of their identity, harmonic relationship, or frequency separation, thus confirming the key role temporal coherence plays in the organization of auditory scenes.

[†]mohsenr@umd.edu
[‡]sas@umd.edu

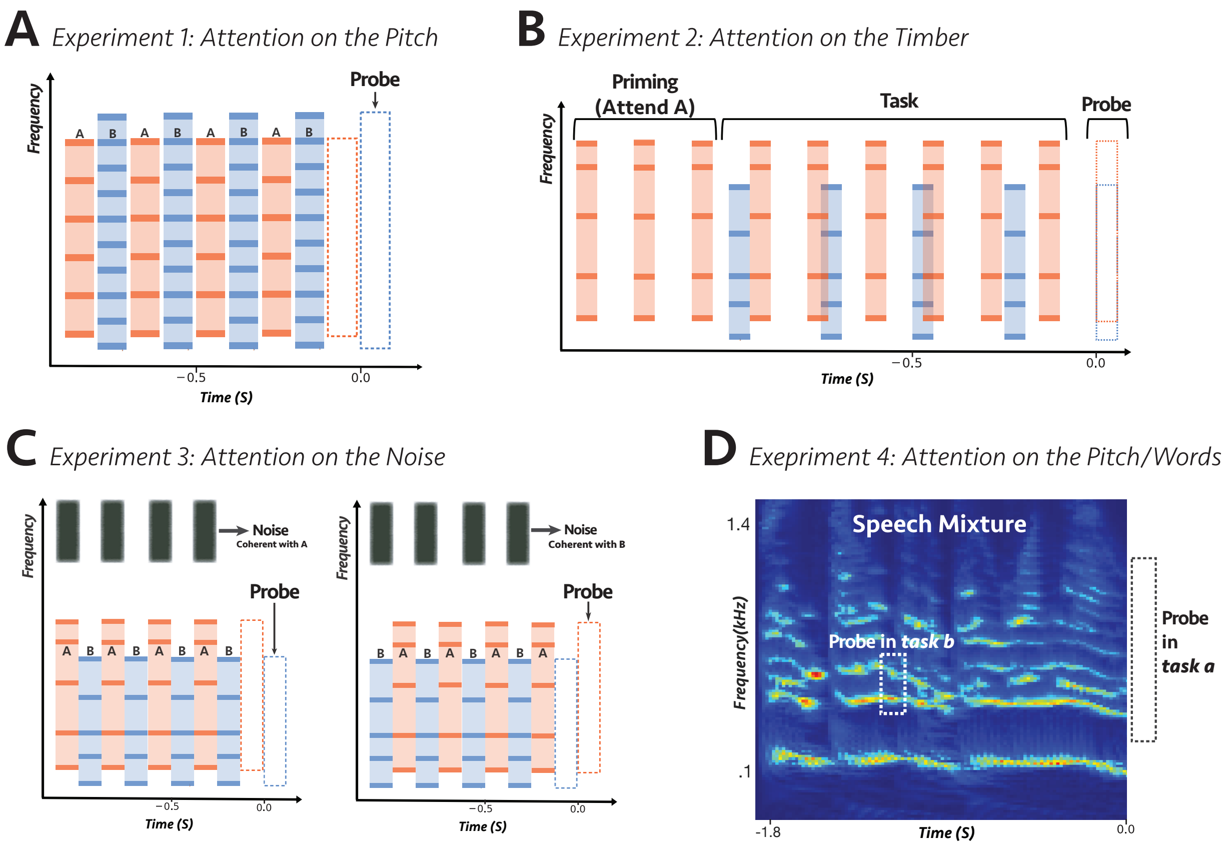


**Figure 1.** ***A summary of stimulus construction in all experiments.*** In all four experiments, the auditory scene consisted of two concurrent auditory sequences. Participants were asked to pay attention to one of the sequences and ignore the distractor. We used an intensity deviant detection task for the first three experiments (panels **A**, **B**, and **C**). In experiment 4, we asked participants to report a specific word in the target voice (panel **D**). To do the task, listeners had to focus their attention on the pitch of the harmonic complex sequence in experiment 1 (panel **A**), attend to the timber of the inharmonic complex sequence in experiment 2(panel **B**), attend to the noise sequence in experiment 3(panel **C**), or pay attention to the target word and pitch of the target voice in experiment 4(panel **D**). We used a probe-tone paradigm (see **Methods**) to investigate the effect of attention on individual frequency components of auditory objects.

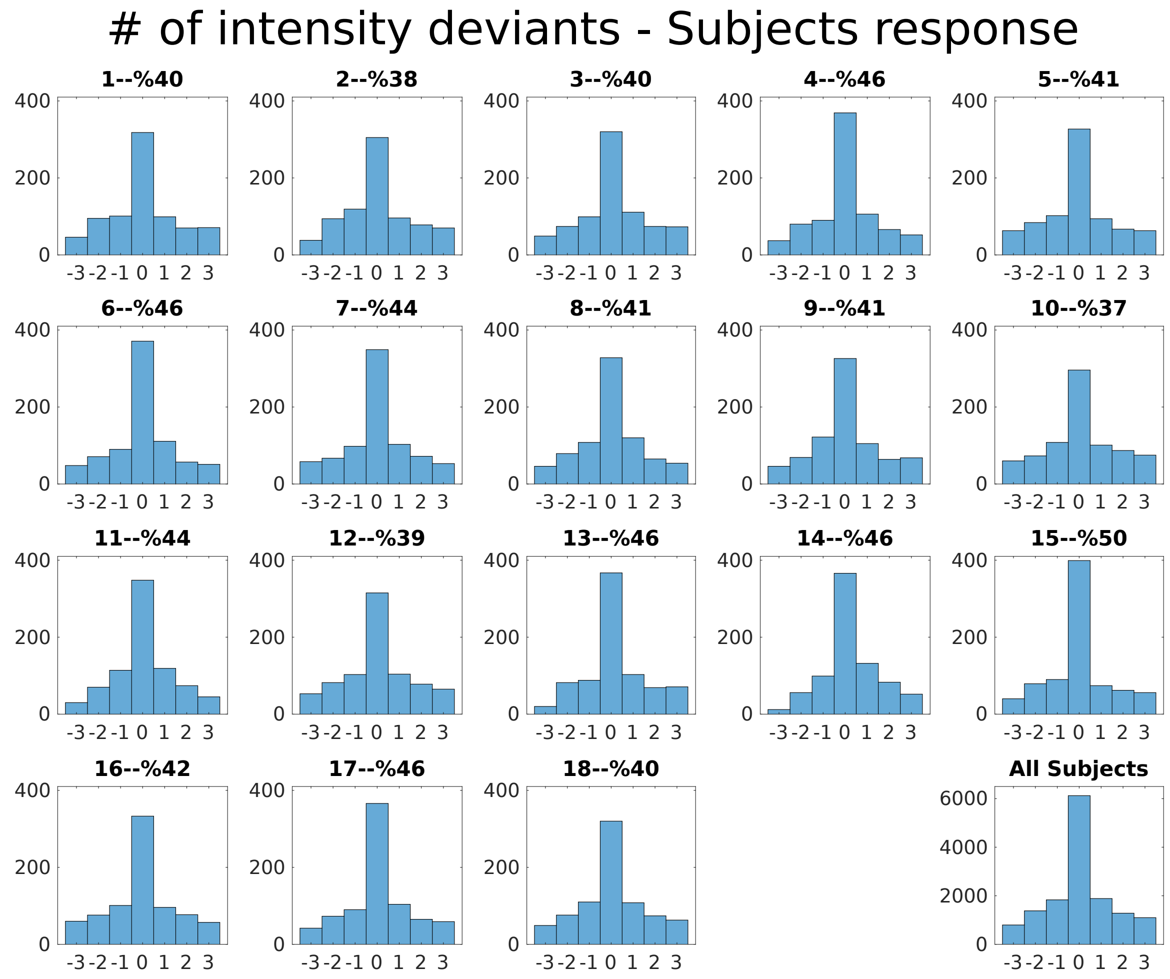


**Figure 2.** ***Behavioral results for experiment 1:*** In this experiment, listeners were instructed to count the number of deviants in the target (attended) sequence, which was uniformly distributed between 0-3 (four choices) across trials, and hence, the chance level was at %25. Each subplot shows the histogram of the true number of deviants minus the subject's response. Therefore, in these subplots, "0" means the correct response (hit), positive numbers mean that listeners missed one or some of the deviants, and negatives mean response was larger than the actual number of deviants. Each subplot's title includes the subject's number followed by their percentage of correct answers (hit rate). All the subjects performed above the chance level.

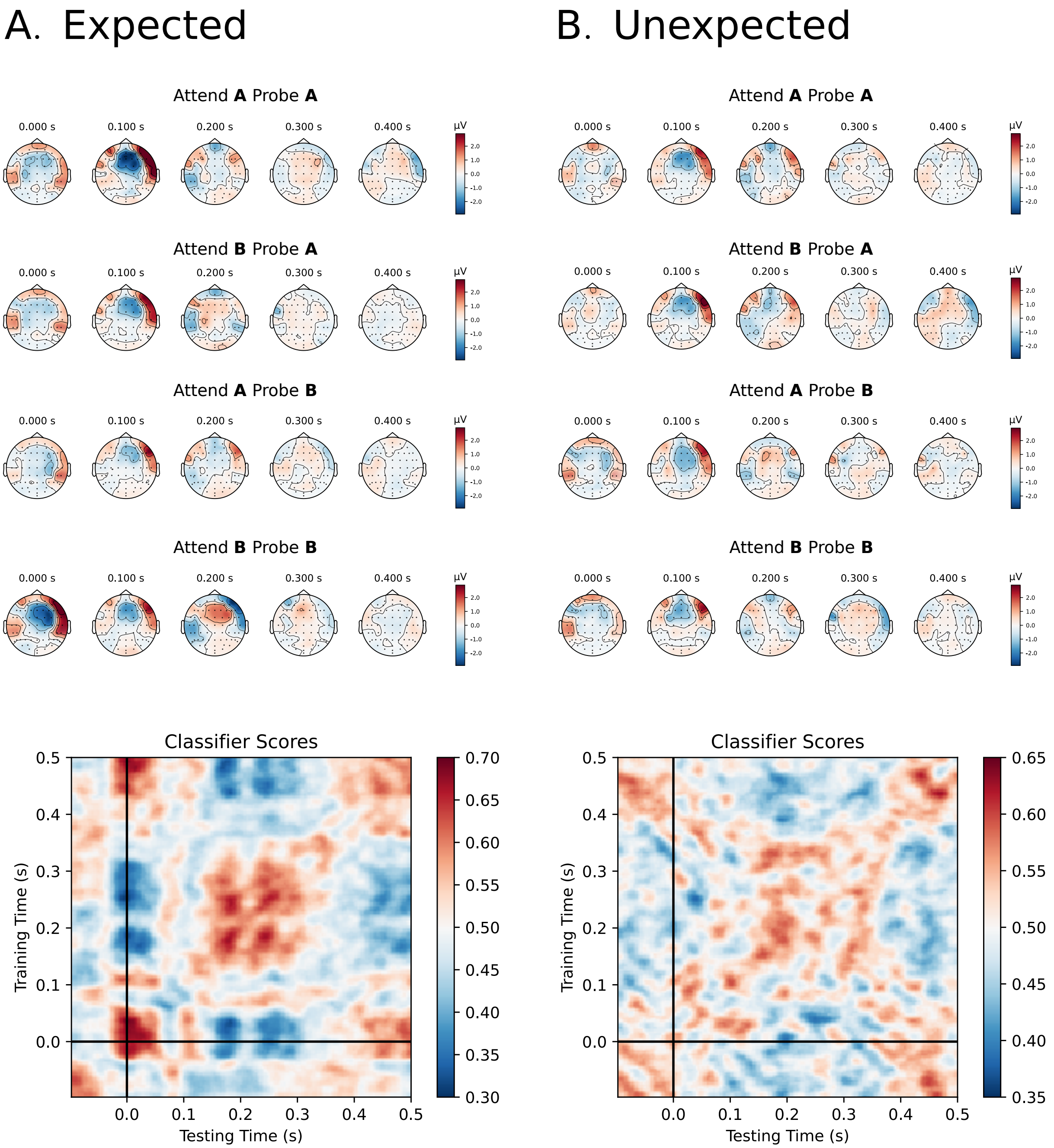


**Figure 3.** ***The relation between the classifier scores and EEG topomaps (a subject example).*** Topomaps of probe A and probe B is plotted for different attentional conditions for **(A)** *Expected* and **(B)** *Unexpected* cases. Linear classifiers were trained at each time point on the responses from all 64 channels (topomaps) in order to decode the focus of attention. At the subject level, the trained classifier could capture the differences in the topomap patterns caused by the attentional changes, e.g., the differences between the topomaps of *Attend A probe A* and *Attend B Probe A*. The classifier scores showed the robustness of the effect for a given subject across all trials; in the second-level test (depicted in **Figure 2B** of the main text), we showed the robustness of the effect sizes across all subjects.

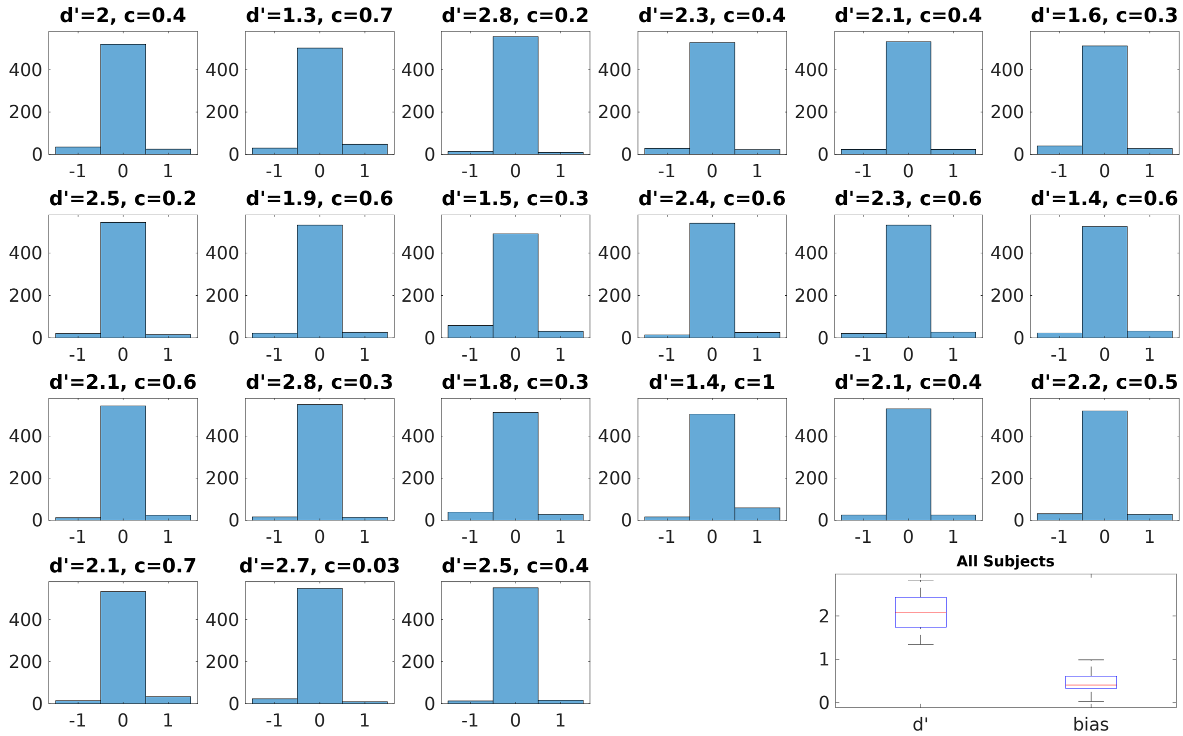


**Figure 4.** ***Behavioral results for experiment 2:*** In this experiment, listeners were instructed to detect a deviant in the target (attended) sequence. Each subplot shows the histogram of a deviant's presence (0 or 1) minus the subject's response. Therefore, in these subplots, "0" means the correct response (hit), +1 means listeners missed the deviant, and -1 reflects the false alarms. The title of each subplot includes the subject's d'prime followed by their bias.

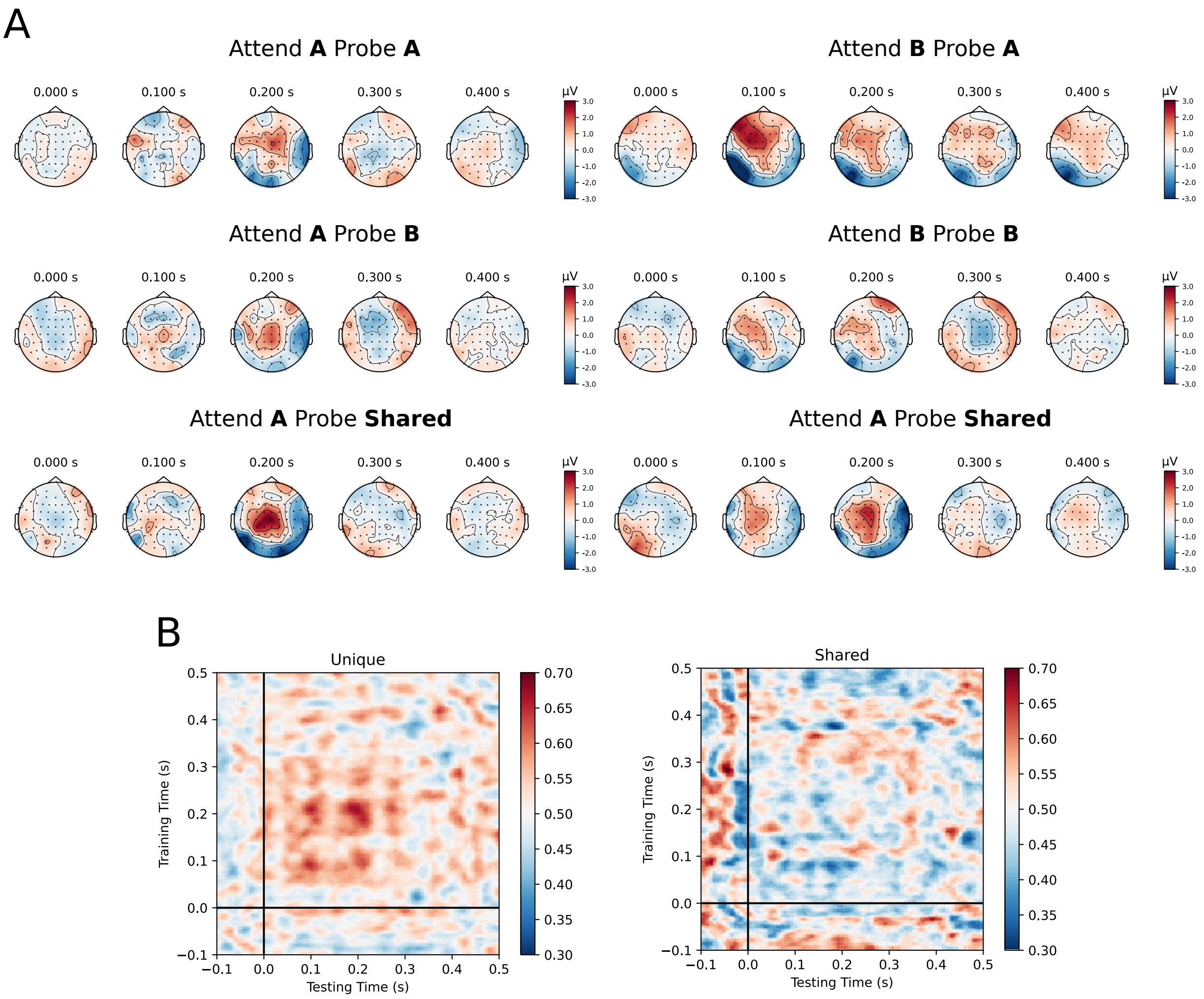


**Figure 5.** ***The relation between the classifier scores and EEG topomaps (one subject example).*** **(A)** Average topomaps of probe A, probe B, and the shared probe were plotted for different attentional conditions. Linear classifiers were trained at each time on the signals from all 64 channels (topomaps) in order to decode the focus of attention. At the subject level, the trained classifier tried to capture the differences in the topomap patterns caused by the attention. **(B)** The classifier scores demonstrated the robustness of the effect for the given subject across all trials. For the unique probe (left), the performance of the classifiers was above chance, which means that there was a consistent difference, i.e., a difference between *"Attend A Probe A"* and *"Attend B Probe A"* in topomap patterns across all trials. Conversely, the shared probe scores suggest that the difference between attentional conditions was not robust since it was not linearly separable.

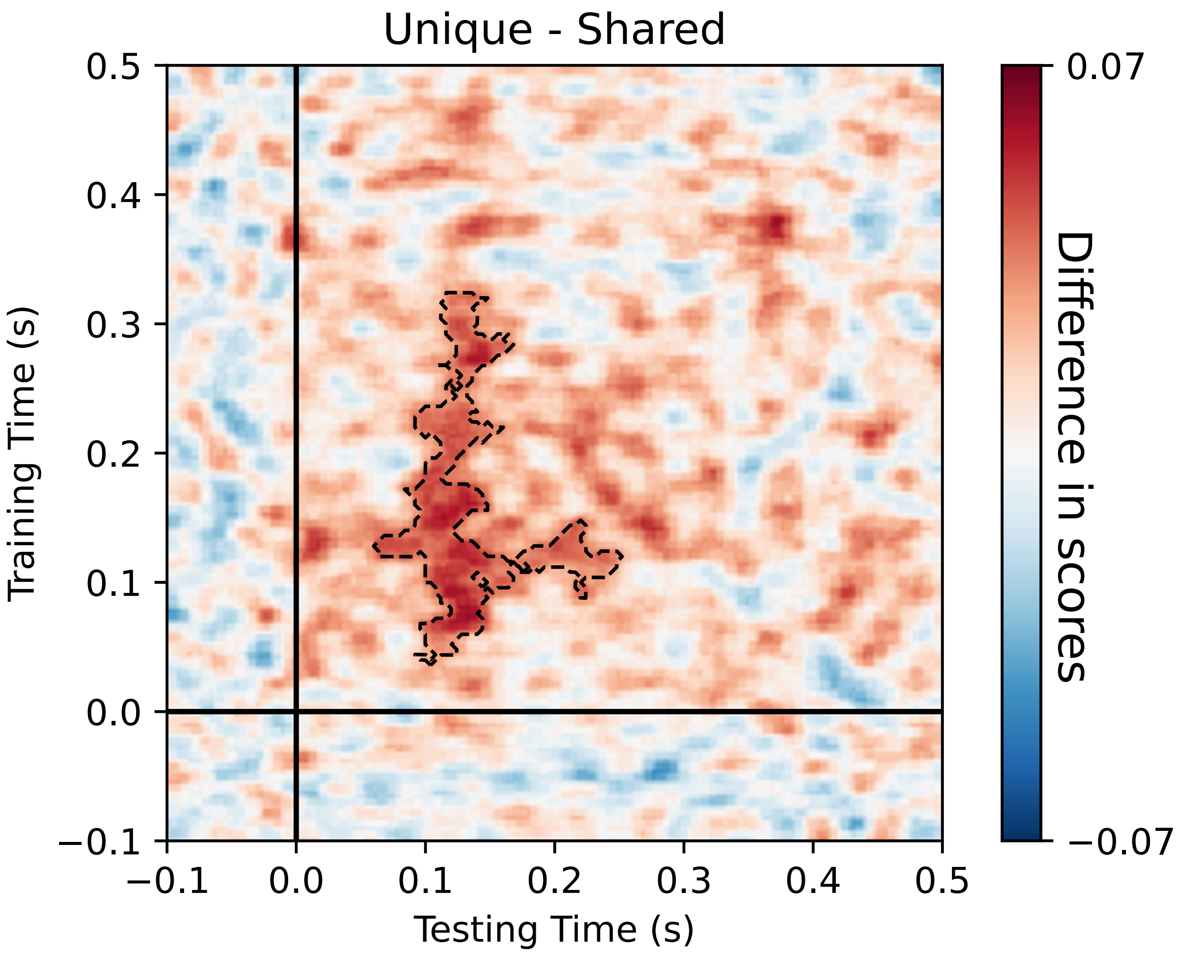


**Figure 6.** ***Comparison between unique and shared decoder scores.*** Scores show the average difference between decoding scores of Unique and Shared probe-tone for all subjects. The difference is significant for the time region contoured between the dashed lines ($p = 0.009$).

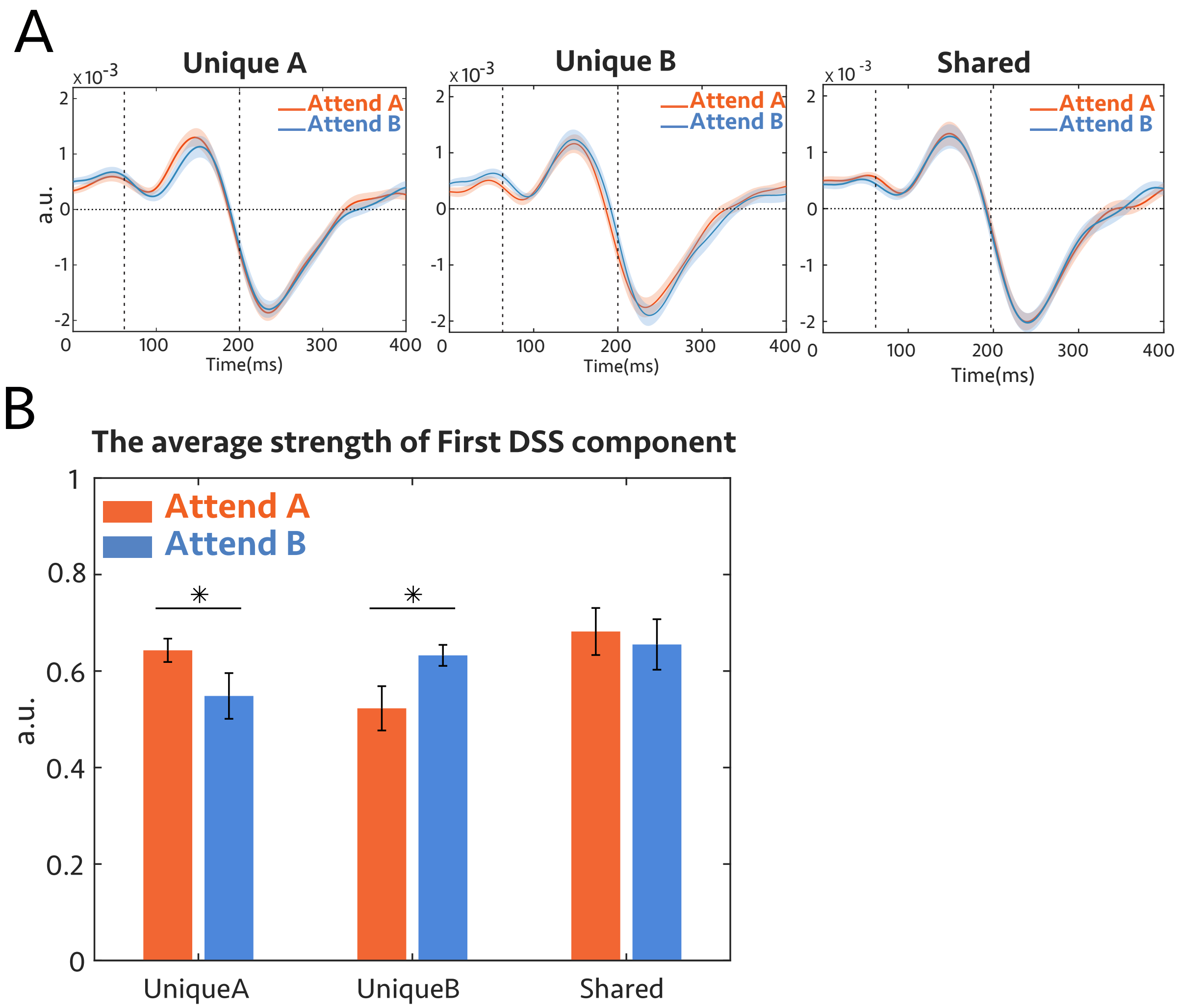


**Figure 7.** ***DSS evoked response.*** Data were submitted to DSS analysis (see Materials and Methods) using the average across trials as a bias filter. The aim was to isolate the most repeatable auditory component by applying a spatial filter. **(A)** Grand average of the most repeatable EEG response to the probe-tone extracted by DSS for each subject; onset of the probe tone is at 0. Left: The response when the probe is at the frequency unique to complex A, middle: when the probe tone is a unique component of complex B, right: The response when the probe tone is a shared component, for attention to tone complex A (orange), and attention to tone complex B (blue). In **Figure 3C** of the main text, we subtracted the orange and blue curves in unique A from the same colors in unique B. **(B)** The *average* amplitude of the neural response from 60 ms to 200 ms after the probe-tone onset. For the unique frequency channels, the attended condition has significantly higher power than the unattended condition ($p = 0.03$ for unique A and $p = 0.01$ for unique B), while the *average* of the shared channel does not show any modulation with attention ($p = 0.6$).

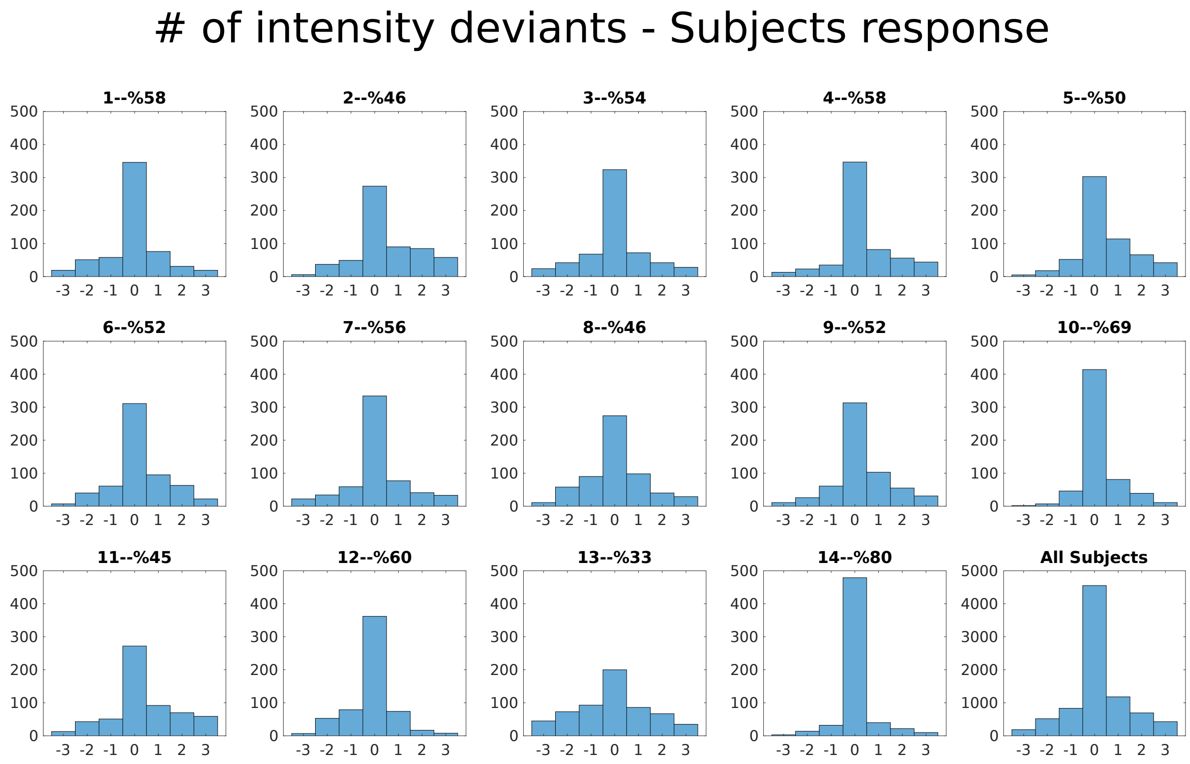


**Figure 8.** ***Behavioral Results for experiment 3:*** In this experiment, listeners were instructed to count the number of deviants in the target (attended) noise sequence, which was uniformly distributed between 0-3 (four choices) across trials, and hence, the chance level was at %25. Each subplot shows the histogram of the true number of deviants minus the subject's response. Therefore, in these subplots, "0" means the correct response (hit), positive numbers mean that listeners missed one or some of the deviants, and negatives mean response was larger than the actual number of deviants. Each subplot's title includes the subject's number followed by their percentage of correct answers (hit rate). All the subjects performed above the chance level (chance level = %25).

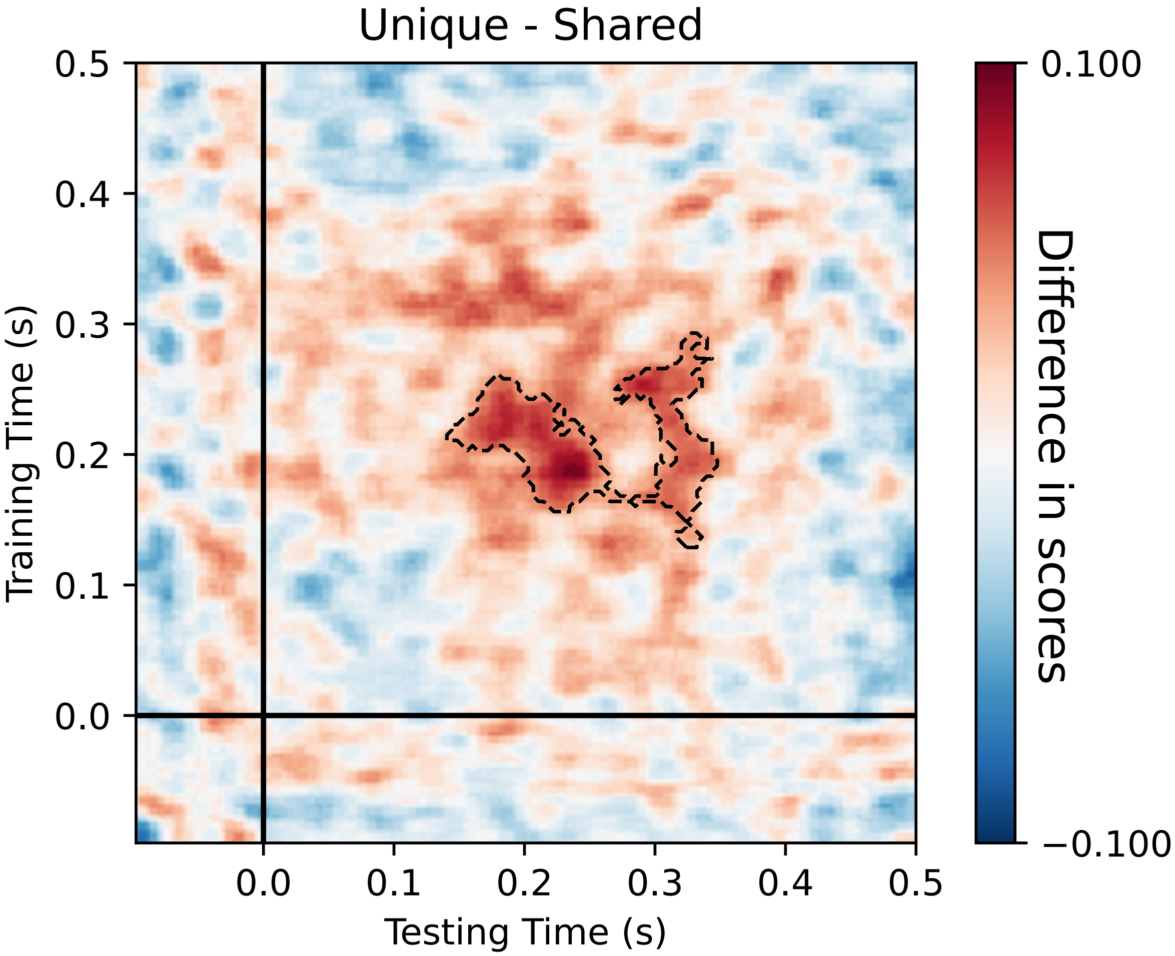


**Figure 9.** ***Comparison between unique and shared decoder scores*** Scores show the average difference between decoding scores of Unique and Shared probe-tone for all subjects. The difference was significant for the time region contoured between the dashed lines ($p = 0.004$).

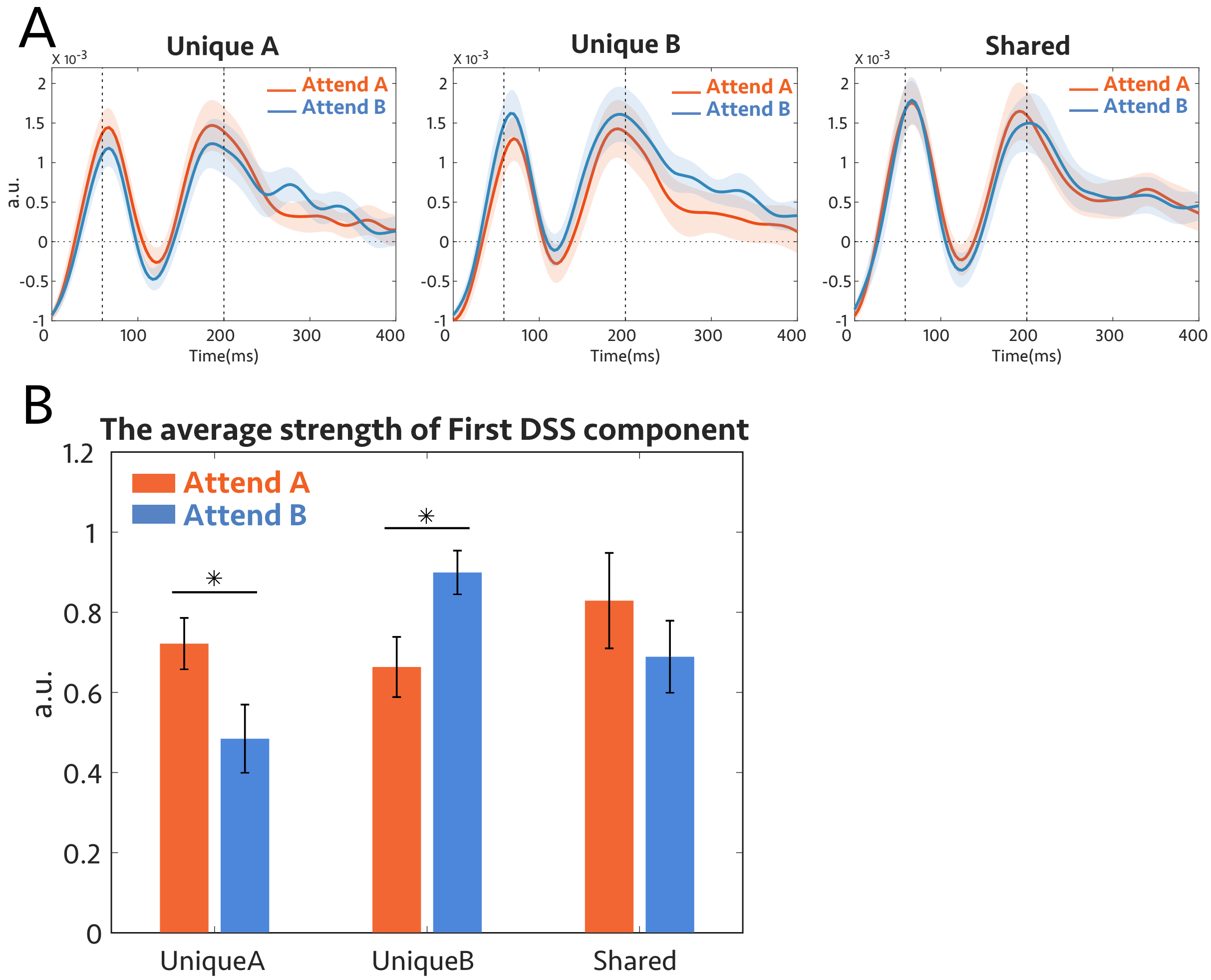


**Figure 10.** ***DSS evoked response.*** Data were submitted to the DSS using the average across trials as a bias filter. The aim was to isolate the most repeatable auditory component by applying a spatial filter. **(A)** Grand Average of the most repeatable EEG response to the probe-tone extracted by DSS for each subject; onset of the probe tone is at 0. Left: The response when the probe was centered at the unique A frequency channels. Middle: The response to the probe-tone unique to complex B. Right: The response when the probe tone was a shared component, under attend to tone complex A (orange) and attend to tone complex B (blue), the curves are comparable. In **Figure 4C** of the main text, we subtracted the orange and blue curves in unique A from the same colors in unique B. **(B)** The average strength of the neural response of the first DSS component from 60 ms to 200 ms after the probe-tone onset. For the unique frequency channels, the attended condition had significantly higher power than the unattended condition ($p = 0.04$ for unique A and $p = 0.01$ for unique B), while the *mean* of the shared channel did not show any modulation with attention ($p = 0.24$).

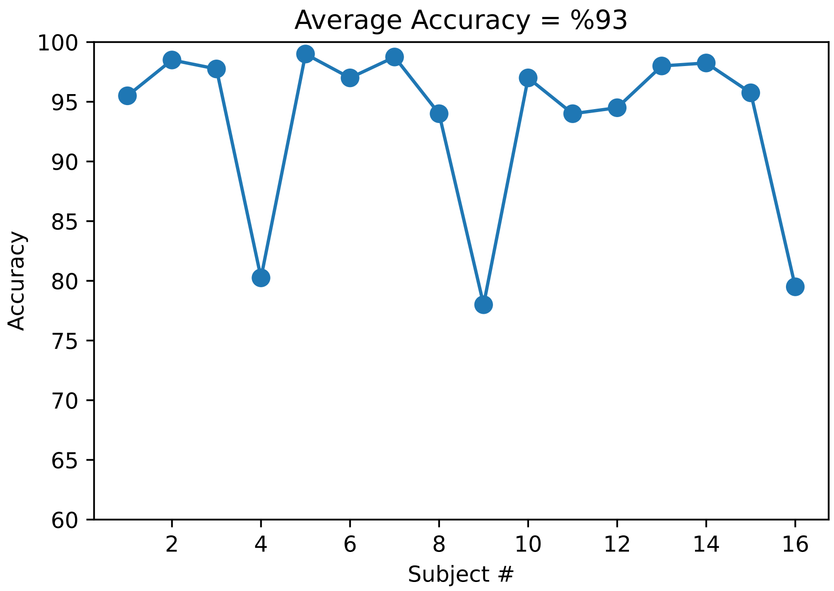


**Figure 11.** ***Behavioral Results for experiment 4a:*** In this experiment, listeners were instructed to report the number or the color of the attended speaker. Each point shows the accuracy for each subject.

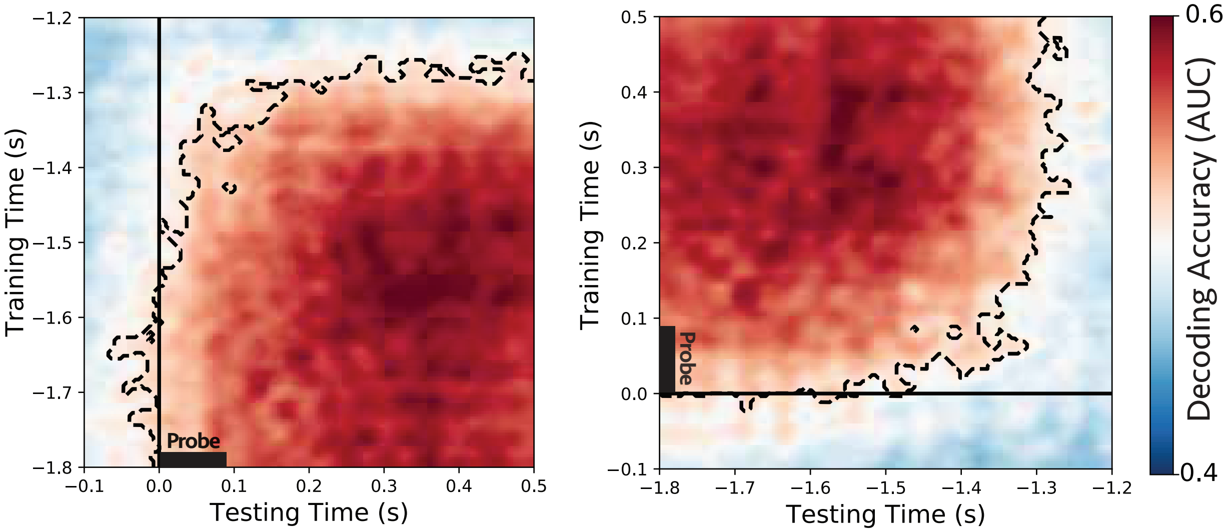


**Figure 12.** ***Generalizing decoders across time.*** Classifiers trained and tested separately at each time instant in two other 600 ms time windows. *Left:* trained at the beginning of the speech (-1.8 sec to -1.2 sec) and tested during the probe-tone (-100ms to 500ms). *Middle:* trained during the probe time window (-0.1 sec to 0.5 sec) and tested during the beginning of the speech (-1.8 sec to -1.2 sec). These results suggest that the modulatory effect of attention is generalizable across times during speech and probe-tone.

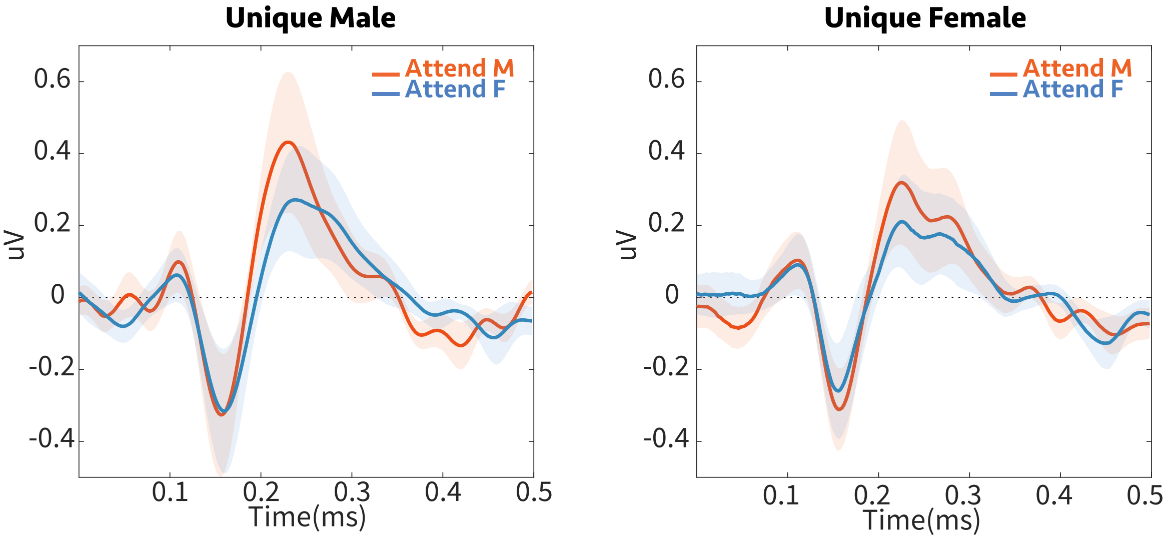


**Figure 13.** ***Evoked responses at channel Cz.*** Average EEG Evoked response to the probe tone at channel 'Cz', computed after denoising data and projecting back the first 5 DSS components to sensor space (see Methods). The onset of the probe is at time 0.- The difference between *unique female* and *unique male* probe tone was computed for the orange (attend male) and blue (attend female) curves for each subject, and it was depicted in **Figure 5C** of the main text.

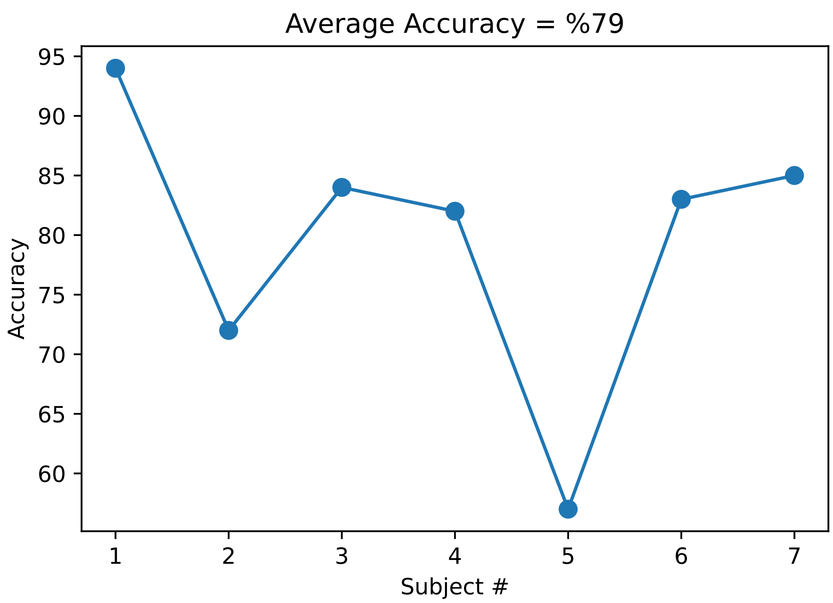


**Figure 14.** ***Behavioral Results for experiment 4b:*** In this experiment, listeners were instructed to report the number or the color of the speaker who uttered the callsign. Each point shows the accuracy for each subject.

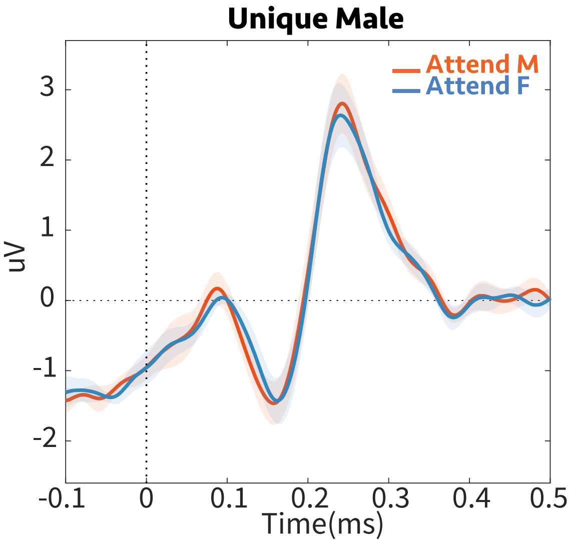


**Figure 15.** ***Evoked responses at channel Cz.*** Average EEG Evoked response to the probe tone at channel 'Cz', computed after denoising data and projecting back the first 5 DSS components to sensor space (see Materials and Methods). The onset of the probe is at time 0. The difference between *unique female* and *unique male* probe tone was computed for the orange (attend male) and blue (attend female) curves for each subject, and it was depicted in **Figure 6C** of the main text.
